# Supplementary figures and images for: SARS-CoV-2 acquisition and immune pathogenesis among school-aged learners in four diverse schools
Source: Pediatr Res. 2021 Jul 24;90(5):1073–80. doi: 10.1038/s41390-021-01660-x (PMC8308070; doi:10.1038/s41390-021-01660-x)

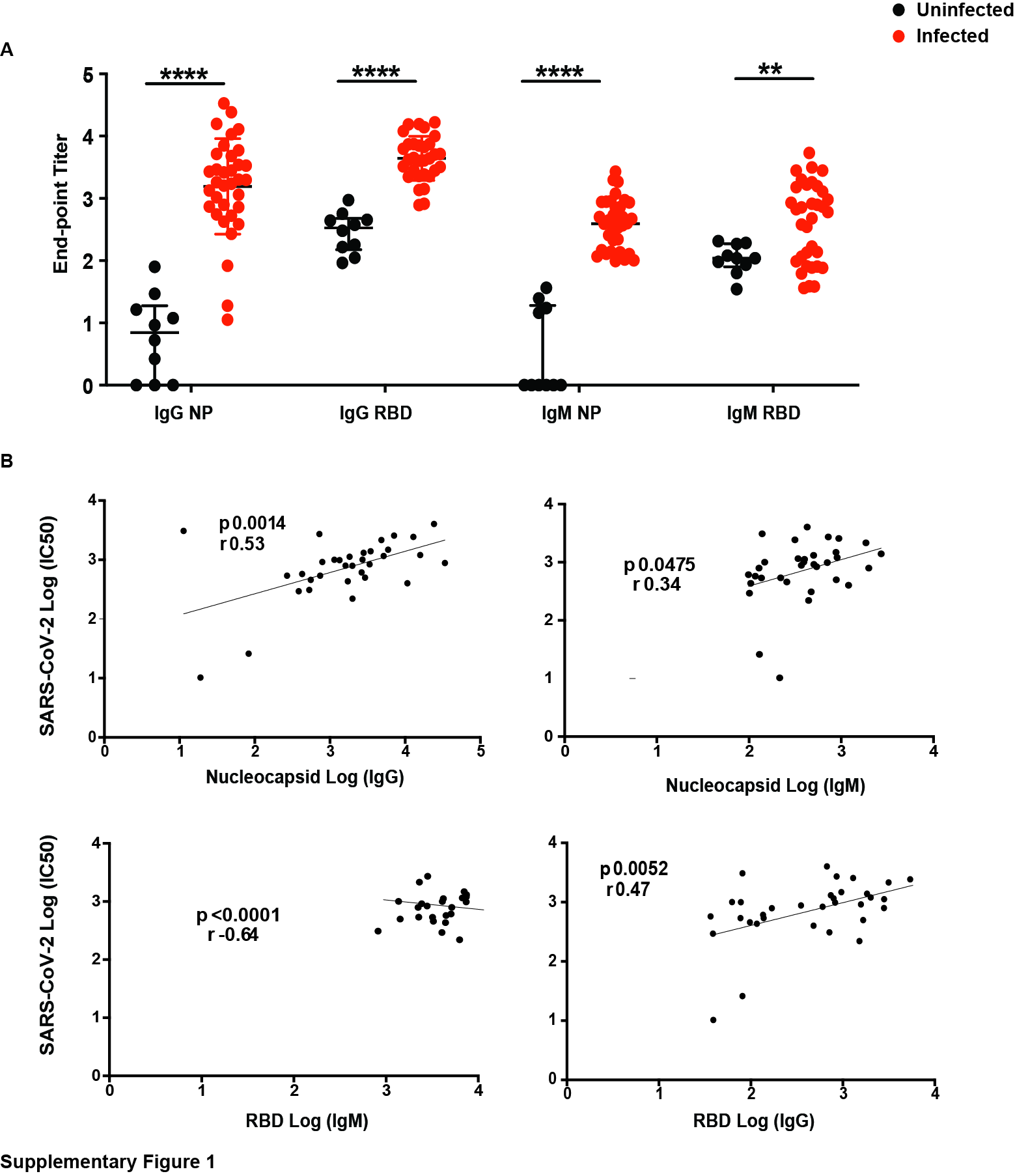

Supplement: Supplementary file 1 — Supplementary Figure 1 [file 41390_2021_1660_MOESM1_ESM.tif]
